# The infant feeding methods promoted by South African Instagram influencers in relation to crying and sleeping, 2018–2020: a retrospective digital ethnography

**DOI:** 10.1186/s13006-023-00555-3

**Published:** 2023-03-16

**Authors:** Sukoluhle Pilime, Sara Jewett

**Affiliations:** Health & Society Division, School of Public Health, University of the Witwatersrand, Johannesburg, South Africa

**Keywords:** Breast milk substitutes, Instagram influencers, Social Media, Crying, Sleeping, Infant feeding, South Africa

## Abstract

**Background:**

Globally, there has been a decline in breastfeeding rates. This has resulted in increased infant mortality due to infectious diseases and inappropriate feeding practices. The aggressive marketing of breastmilk substitutes (BMS) by manufacturers has contributed, in part, to these declines. With the progressive use of social media, marketing has shifted from traditional methods to the use of influencers, who command a huge following on their social media accounts and influence the daily decisions of their followers. This study investigates the infant feeding methods and associated products promoted by South African influencers in relation to crying and sleeping and their followers’ responses.

**Methods:**

This was a retrospective study, which used a mixed methods digital ethnographic approach to analyse posts related to infant feeding methods that were made by seven South African Instagram influencers between the period of January 2018 to December 2020. Framing analysis was used to analyse qualitative data and quantitative data were analysed descriptively.

**Results:**

From the 62 posts that were analysed, 27 were sponsored advertisements (some violating local regulations) and 35 posts promoted breastfeeding. The 18,333 follower comments and 918,299 likes in response to the posts were also analysed. We found that influencers presented BMS products as a solution for a child who cries a lot and has trouble sleeping. BMS were framed as helpful for children who are seemingly always hungry and dissatisfied with breastmilk alone. The study also found that some influencers promoted breastfeeding on their Instagram pages. Unlike BMS posts, breastfeeding posts were not sponsored. With a few exceptions, followers tended to support and reinforce the framing of influencers.

**Conclusion:**

Stiffer regulations should be enforced against companies using influencers to promote infant formula and other BMS products, with proactive monitoring of social media. Professionals giving advice contrary to the guidelines from the WHO should be reported according to Regulation 991 and made accountable. Proactive engagement with Instagram influencers to promote breastfeeding should be considered.

## Background

There are many studies that highlight the importance of breastfeeding in maternal and child health. Some noted advantages include protection against diarrhoeal diseases, improvements in the lifelong health of the child, psychosocial and socio-economic benefits for both the mother and the child and significant reduction in infant morbidity and mortality [1, 2]. In South Africa, breastfeeding promotion is a national health priority [3]; however, the country still has sub-optimal breastfeeding rates [4]. For children under six months of age, the estimated exclusive breastfeeding (EBF) rate for infants between 0–1 month it is estimated to be 44%, but declines to 24% for infants between 4 -5 months [4].

Problems with excessive crying and sleeping are found in approximately 20% of children and have been known to inform the parental decisions for alternate infant feeding methods [5]. Caregivers associate constant crying with inadequate breast milk production and, in addition, an infant who seems continuously hungry motivates the initiation of alternate feeding methods [6]. Caregivers’ perception of infant fussiness, posseting and sleep as “problematic” also shape their infant feeding practises, often resulting in breastmilk substitute (BMS) introduction [7].

**Fig. 1:**
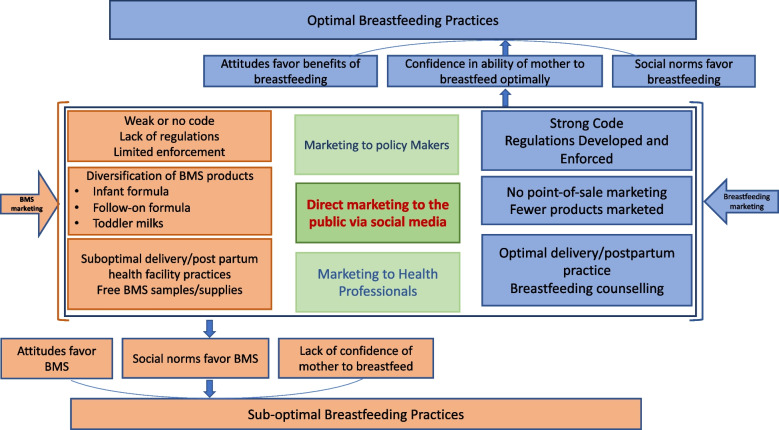
Adapted conceptual framework of the impact of marketing of BMS on WHO recommended breastfeeding practices [10]

Companies that produce BMS market their products as solutions for infants who struggle with the abovementioned problems [8]. Partly as a result of their aggressive advertising tactics, breastfeeding rates have significantly dropped globally [9,10]. The conceptual framework shown in Fig. 1 is from a published study on the impact of BMS marketing on WHO recommended breastfeeding practices [10]. The framework illustrates the impact that different forms of marketing have on the decision to use BMS or to breastfeed. The review found evidence that BMS marketing influences social norms and attitudes, erodes the confidence of mothers to breastfeed and results in sub-optimal feeding, although they struggled to quantify how different marketing strategies contributed to these patterns. The review also did not cover social media marketing and only focused on commercial infant formula. As such, we have adapted the figure to indicate our interest in social media as an additional form of direct marketing. Of interest to this study is direct marketing of either BMS or breastfeeding to the public through social media influencers.

In response to the marketing practices of BMS manufacturers and the subsequent increase in infant mortality rates, the International Code for the Marketing of Breastmilk Substitutes was developed by the World Health Assembly (WHA) in 1981, herein referred to as the Code [11]. The Code has various articles and subsequent resolutions to regulate how governments, health systems and health care workers provide guidance relating to Infant and Young Child Feeding (IYCF). The Code also monitors responsible marketing and labelling of BMS by manufacturers [10].

Drawing from the precepts of the Code, in 2012 South Africa developed its own Regulations Relating to Food Stuffs for Infants and Young Children (R991) to regulate IYCF practices [12]. In terms of R991, there are restrictions placed on the labelling and marketing and promotion of infant follow up formulae, and powdered or liquid milk being represented as suitable for infants and young children [13]. Complementary feeding bottles, feeding cups and teats are also implied by the Code and the local R991 [14]. The marketing of BMS and products is prohibited for infants under six months of age and promotional practises of certain ‘designated’ products (for children under 36 months) contravenes the Code and R991.

**Fig. 2:**
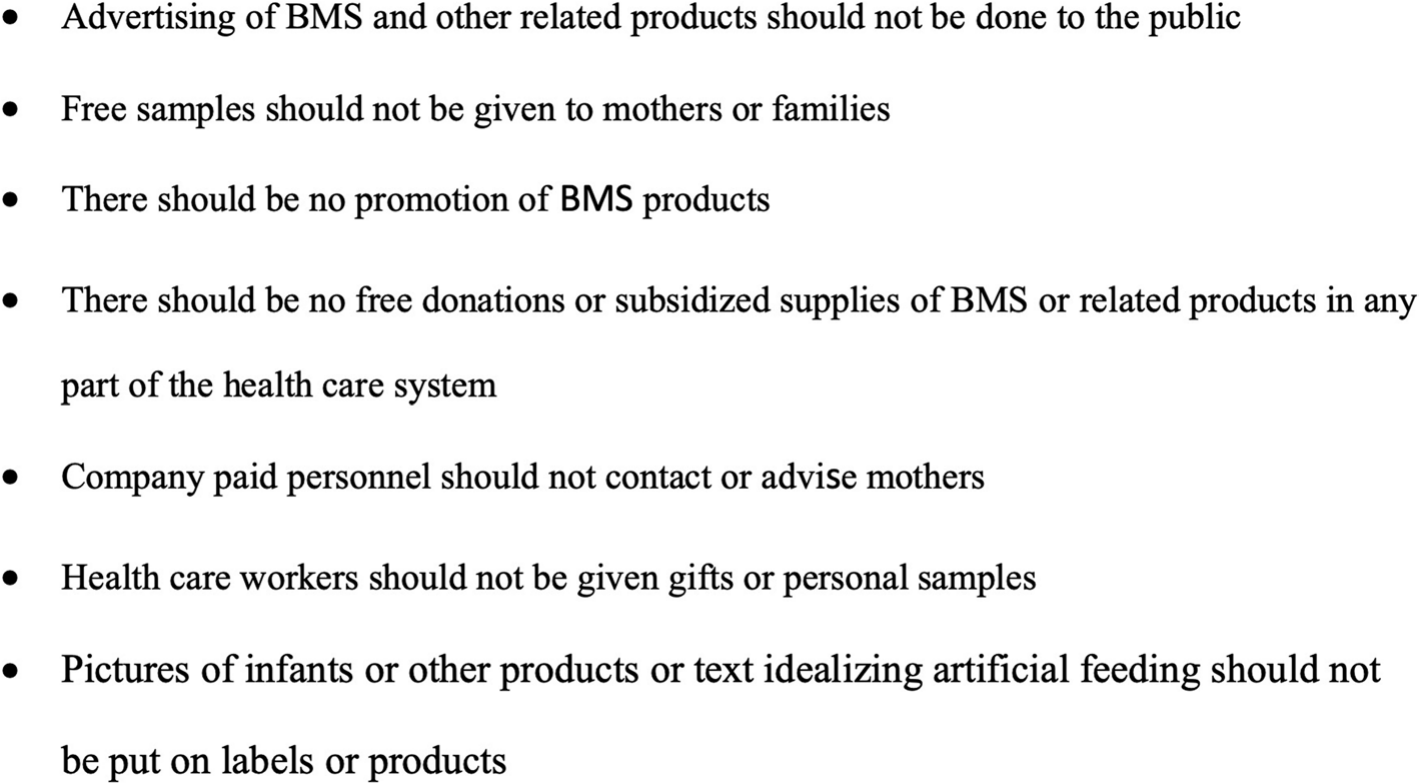
Key elements of Regulation 991

Certain country-specific regulations have expanded the definition of BMS to include pacifiers, for example Vietnam [15], but this has not been the case for South Africa. It is important for more countries to consider expanding their definitions of BMS to include pacifiers as there are risks associated with pacifier use. These include failure of breastfeeding, dental deformities, sleep disorders, tooth decay and oral ulcers [16].For this study, we monitored the marketing of pacifiers within our working definition of BMS, but did not list these as R991 contraventions (see Fig. 2 for key elements of R991).

Despite the Code and country-specific regulations like R991, research has shown that companies are increasingly using the internet and social media sites as well as mobile applications to support and sell their BMS products [17]. Studies conducted by the WHO across different countries found that companies producing formula milk access personal data and engage with women through online platforms, which optimizes their marketing methods [8]. This is relevant, as in South Africa, almost 30 million out of an estimated 57 million people are active social media users [18]. With this wide usership, companies are now marketing their products on various social media platforms through the use of social media influencers [19]. Influencers are people who command a following on their social media profiles, separated into different categories based on their number of followers. We applied the following category definitions of mega influencers (more than one million followers), macro influencers (between 40,000 and 1 million followers), micro influencers ( between 1,000 and 40,000 followers), and nano influencers (less than 1,000 followers) on one social media platform [20]. We note that other social media scholars assign different thresholds, such as a macro influencer having between 100 K and 1 million followers and a nano influencer being anyone with less than 10,000 followers [21], and that these appear to be somewhat arbitrary. Whatever their following sizes, individuals called influencers have developed credibility from their followers owing to the content that they post [22, 23], with some scholars arguing that there are times the micro-influencers are preferable to macro-influencers [24]. One way that influencers are identified on social media platforms is through having a verification badge or a “blue tick” next to their usernames [19], although this is not a pre-requisite and recent shifts away from this model on Twitter may have ripple effects elsewhere. Influencers can vary from celebrities, to people who are not necessarily celebrities but have acquired followings from the content that they post, thereby gaining popularity on the social media platforms and making careers out of marketing for various companies on social media [22, 23]. The majority of influencers in South Africa are female (56.2%) and they are aged between 18 and 34 years old [25].

Drawing from the work of Piwoz and Huffman, and based on evidence of the growth of social media marketing in South Africa [26], this study sought to answer the following research question: How are South African Instagram influencers marketing infant feeding in the context of infant and young child feeding regulations in South Africa? Our first objective was to investigate how South African Instagram influencers presented BMS to parents and caregivers with regards to crying and sleeping during the first two years of an infant’s life, in light of R991 regulations. To support contextual comparison, we also looked at breastfeeding mentions by Instagram influencers during the same study period, a method promoted by Macnamara for media content analysis [27]. Beyond frequency of mentions, our second objective was to explore how infant feeding options were framed, particularly by influencers, but also by the followers who engaged with these posts. The third study objective was to explore the conversations on infant feeding between the selected influencers and their followers.

## Methods

### Study design

In this study we used a retrospective digital ethnographic approach [28], which involved collection of behavioural data of participants in their natural real life settings without the use of questionnaires, whereby what they say and what they do can be vastly different [29]. The ethnographic approach was done online. As researchers, we observed the interactions between the influencers and their followers without interference. We studied the Instagram posts and associated images using a mixed methods approach, drawing on both qualitative and quantitative data to address the study objectives.

### Study setting

Instagram was selected as the social media platform of choice because in South Africa about 6 million people are Instagram users and the majority (54.2%) of these users are female aged between 18–34 years old [30]. We focused on posts originating from South African Instagram influencers. Given the nature of social media, responses to posts may have originated from anywhere.

### Study population and sampling strategy

The study population was all posts by South African Instagram influencers targeting mothers/parents and caregivers of infants that discussed infant feeding methods for the first six months of life during the study period of January 2018—December 2020, and subsequent engagements (comments and likes) on these posts. The search strategy was designed to identify infant feeding posts advertising BMS, which is defined as any foods or liquids that are marketed or presented as a total or partial replacement of breast milk [11] as well as breastfeeding. There was no requirement that the posts be overtly sponsored, given that disclosure is not ubiquitous and regulations vary by context [31]. This timeframe was selected so that the most recent data at the time of writing up the research would be presented. As there was not a central repository that defines influencers to act as a sampling frame, identification of eligible influencers and then eligible posts was done as follows:

- Step 1. South African Instagram Influencer Identification.

The authors manually searched for South African influencers who had children and posted content related to having babies in South Africa on the Google search engine using the following Boolean search terms: Influencers AND South Africa; Celebrity AND Influencer AND Mom AND SA; Influencers AND Babies AND South Africa; and, Influencers AND Moms AND Babies AND South Africa. For each term, both authors reviewed the results from the first two pages of results and from these results selected the URLs and associated Influencer names and handles to confirm that they were South African. Seven influencers that met inclusion criteria were identified at this stage.

After selecting the influencers to be analysed in the study, the first author clicked the follow button on their profiles in order to access their posts as they were shared for the entire study period. While it is possible to access posts without having formally followed the influencers, as their profiles are publicly accessible, for the purposes of this study, the researcher followed the pages of the influencers to get real-time access (access as content is shared) to ensure posts were not missed. This approach is similar to the methods of a study that was conducted in South Africa on the marketing done by companies producing BMS on social media but without using influencers [26].

- Step 2. Eligible post identification.

We went on the Instagram handles of the selected influencers to search for posts that fit into the post eligibility criteria for inclusion in analysis. The eligibility criteria were as follows:

- The post and subsequent comments (for inclusion) should have been made between January 2018 to December 2020.
- The post must be publicly accessible
- Post must address infant and young child feeding practices
- Step 3. Data extraction.

The first author downloaded the eligible posts and their subsequent comments, number of likes, link, caption and username of the influencer and saved this in an Excel database. To download the videos, we copied the links of the video posts and pasted them on a link [32], and then stored the downloaded videos in an Excel database for later analysis. To download the comments, we entered the URL from the posts on a link [33] and then pressed the scrape button. All the comments were downloaded into a file and then saved in an Excel database. Follower identities were not included to protect their identities, as they are not influencers.

### Data processing and analysis

The final sample of collected data included 62 eligible South African Influencer posts from the seven influencers, comprising 61 images, one video and 18,333 comments (both influencers and their followers). The images, influencer and follower comments were imported into NVivo12 for qualitative framing analysis, while descriptive quantitative data were coded manually in Excel.

Table 1 summarises the dimensions of the posts that the authors agreed upon jointly, prior to manual coding for the quantitative analysis. The first author did the entire quantitative coding. Influencer posts were coded for all dimensions in the table whereas only the dimension of Slant was applied in relation to follower responses. The follower responses were categorised as pro-breastfeeding (indicating follower comments supporting breastfeeding), pro-BMS (follower comments supporting BMS and co-feeding of infants 6 months and younger)and neutral, and the findings were descriptively analysed, as in Table 2. For quality purposes, partway through the coding, the second author independently coded a random sample of posts. There was full agreement with the first author, analogous to an interrater reliability of 100%. This high level of agreement is likely to have developed through a previous study where the authors used a similar coding framework to analyse BMS marketing in magazines [34].

**Table 1:** Dimensions captured for quantitative analysis

**Infant feeding**	# of posts discussing infant feeding methods for infants by the influencers fitting the inclusion criteria: EBF for infants < 6 months, breastfeeding up to 36 months, complementary feeding, BMS use
Crying	# of posts discussing BMS in relation to crying on the influencers’ handles
Sleeping	# of posts discussing BMS in relation to sleeping on the influencers’ handles
Ads	# of adverts by companies producing BMS on the selected influencers’ pages. Adverts are posts made by influencers on behalf of companies, and they usually get paid for the posts
Sources	who was quoted in the posts as giving advice on infant feeding, e.g. categories of health care professionals, mother to mother, BMS producing companies’ representatives
Slant	Attitudes towards breastfeeding or BMS of both original posts and follower responses: Pro-BMS, Pro-breastfeeding, neutral
Violations Sponsorship	# of posts potentially violating R991 and the Code # of posts that an influencer was paid to share on their profile. These posts are identified through phrases such as “paid partnership with”, “#AD, #sponsored by” etc
Type of BMS	BMS broken down by type, ie bottles/teats, gadgets for facilitating the use of BMS, pacifiers, complementary food marketed for infants younger than 6 months and baby formula

**Table 2:** Follower engagement for each influencer’s infant feeding (IF) posts

Influencer (Instagram handle)	Number of followers^a^	Influencer category	Number of IF posts^a^	Number of likes^a^	Number of comments^a^
	**N**		**n**	**n**	**n**
1.**Nandi Madida** (Nandi_Madida)	2,700,000	Mega Influencer	1	92,700	730
2.**Jessica Nkosi** (jessicankosi)	2,600,000	Mega Influencer	1	11,700	137
3.**Mpoomy Ledwaba** (Mpoomy_ledwaba)	370,000	Macro Influencer	4	181,923	1,023
4.**Ntando Kunene** (kunene_ntando)	361,000	Macro Influencer	1	n/a	24
5.**Nkateko Dinwiddy** (takkies7)	347,000	Macro Influencer	20	498,743	15,685
6.**Keke Mputhi** (kekemputhi_official)	211,000	Macro Influencer	3	394	394
**Totals**	**6,790,000**		**62**	**924,396**	**18,333**

^a^The source for number of posts, followers, likes and comments was the Instagram influencer handles, collected in December 2020

For the qualitative aspect of the study, the first author viewed each image/video and analysed the subsequent captions and comments that followed each post using framing analysis [35]. This analysis focused on how influencers structured the delivery of information regarding infant feeding according to their own experiences and, in other cases, how this was structured according to what they were promoting. For the first round of coding, the first author went through the Influencer posts, captions and follower comments and categorizing them into themes, which she discussed with the second author, using examples. Framing analysis was done on the most prominent themes, as agreed upon by the co-authors. Throughout the framing analysis, we jointly explored how rhetoric, analogies and metaphors in common language were used to promote or undermine the use of BMS as well as breastfeeding.

Given the interpretive nature of framing analysis, it is important to specify our training and positionality as researchers. Firstly, both authors are trained researchers drawing on a constructivist epistemology for qualitative analysis. As public health practitioners, we approached analysis as supporters of breastfeeding, grounded in an understanding of local BMS regulations. However, we also drew on our experiences as mothers who breastfed, but also relied on commercial infant formula in some instances. Our training alongside our personal experiences enabled us to discuss influencers and followers in terms of their interface with science and regulations as well as from the perspective of what frames might appeal to parents who have difficult infant feeding journeys.

In order to enhance the credibility of the research, triangulation, through cross checking the posts and comments, analysis of data collected as well as auditing the data for consistency was done by both authors. To enhance reliability of the study, the second author reviewed a sample of posts and checked the way the first author was coding the qualitative comments, similar to what she did with the quantitative coding. There was 100% inter-coder reliability. Furthermore, the researchers discussed and revised any inconsistencies.

## Results

### South African influencer reach

A total of 62 Instagram posts that were influencer-initiated and met the inclusion criteria were identified for the period of January 2018 to December 2020. As shown in Table 2, the seven influencers included in the analysis had a total number of 6,790,000 followers, representing their total audience. Two were mega-influencers, with well over 1 million followers, while three were macro-influencers and the remaining two were micro-influencers. In addition to the original posts, data were collected on the response and engagement of the followers through the number of likes and the number of comments each post received. Cumulatively, the 62 eligible posts received 918,299 likes and 18,333 comments, which were analysed over a three-month period in 2020.

There were no likes on Ntando Kunene’s video because she posted this on her stories, and posts made on this feature do not have a ‘like’ button. Therefore, it was not possible to establish how many people would potentially react to the video through likes.

### Influencer infant feeding focus

Of the total eligible posts by influencers that were analysed, 43% were advertisements promoting products by companies producing BMS (specifically, NUK, Phillips Avent and Ella’s Kitchen) and 57% of the posts promoted breastfeeding. There was no explicit endorsement of formula feeding. However, indirect facilitators of formula, such as feeding bottles were advertised by two of the three companies. Gadgets which are used to facilitate early mixed feeding (under six month of age), for example baby food makers and squeeze stations, were also advertised. Of the posts promoting breastfeeding, 43% were made during breastfeeding week, which is held from the 1^st^ to the 7^th^ of August every year.

As shown in Table 3, four of the influencers posted specifically about BMS, with some potential violations to Regulation 991 recorded. There were six influencers who referred to breastfeeding during the study period and three influencers referred to both BMS and breastfeeding. Among those influencers who posted both BMS and breastfeeding posts, no clear pattern could be established in terms of the number of times they posted the different infant feeding methods. For example, macro-influencer Azwi Rambuda had a total of 23 sponsored IF posts and 9 non-sponsored breastfeeding posts, while another macro-influencer, Nkateko Dinwiddy, had only 1 sponsored BMS post and 19 non-sponsored breastfeeding posts.

**Table 3:** Types of posts by the influencers and potential Regulation 991 violations

Influencer	Sponsored BMS Posts	Non-sponsored Breastfeeding posts	Potential violations of R991	Total Infant Feeding posts
	**n**	**n**	**n**	**n**
**Azwi Rambuda**	23	9	22	32
**Mpoomy Ledwaba**	2	2	2	4
**Nkateko Dinwiddy**	1	19	1	20
**Jessica Nkosi**	1	0	0	1
**Ntando Kunene**	0	1	0	1
**Nandi Madida**	0	1	0	1
**Keke Mputhi**	0	3	0	3
**Total**	**27**	**35**	**25**	**62**

Table 4 indicates the specific type of BMS advertised by the influencers. There were no posts advertising baby formula, and only one post was an advert for complementary food, directed at infants less than six months of age. Most of the advertising was done for gadgets that facilitate the use of BMS. This could be attributed to awareness by the manufacturers of the implications of illegal advertising but still finding ways to advertise that are seemingly less evident.

**Table 4:** Post by specific type of BMS

BMS type	Number of posts
Bottles/teats	16
Gadgets for facilitating the use of BMS	9
Pacifiers	1
Complementary food marketed to infants less than six months	1
Baby formula	0

### Influencer framing of BMS in relation to crying and sleeping

The methods and language used by influencers to describe infant feeding, particularly BMS, was of particular interest in terms of frames related to crying and sleeping. Table 5 highlights the breakdown of the posts in relation to crying or sleeping for those influencers who posted about BMS. Only two influencers linked BMS to these issues. Specifically, Azwi Rambuda discussed complementary feeding to help infants with sleep and to address crying infants, while Jessica Nkosi referred to a pacifier as a solution for a “fussy baby”. The other frames they discussed, for example bottles and teats mimicking the breast are outlined in the qualitative analysis that follows.

1. *“Pacifiers soothe crying babies”*

**Table 5:** Frequency of BMS mentions in relation to crying or sleeping, by influencer

Influencer	BMS posts	Crying	Sleeping	Other*
	n	n	n	n
Mpoomy Ledwaba	2	0	0	2
Azwi Rambuda	23	1	2	20
Jessica Nkosi	1	1	0	0
Nkateko Dinwiddy	1	0	0	1
**Totals**	**27**	**2**	**2**	**23**

BMS manufacturers used South African influencers to spread the narrative that pacifiers are a solution to a child who cries a lot or is fussy. In the instance highlighted below, an influencer posted an image of a Phillips Avent pacifier and framed it as the best alternative in the market that helps with a child who cries a lot.

*The first few weeks of Sedi’s arrival, boy, he’d cry. Pretty much every second, ok, maybe not second, you do catch my drift though. Had to come up with different ways to soothe him, and a Paci was it. Not just any Paci but the smooth Philips Avent Paci. He had it for approximately four months, and in those months he slept peacefully, the crying was reduced and mommy rested too.* (Azwi Rambuda)

Jessica Nkosi made the same claims about pacifiers soothing crying or ‘fussy’ babies, advertising a different brand of pacifiers called NUK. Both of these companies market BMS products, namely bottles and teats, which are covered by R991.

2. *“Bottles and pacifiers mimic the breast”*

Another pattern in the framing of company-sponsored posts was the use of persuasive language to sway followers into using bottles or pacifiers, through portraying them as equal to the breast. In a Phillips Avent sponsored post, an influencer uploaded an image of a Phillips bottle with a caption describing how breastfeeding is difficult for a working mom, for the baby and the caregiver. The influencer described how the Phillips Avent bottle was the ideal solution because it mimics the breast and is ideal for combination feeding. The post explained that by using the Phillips bottle, there was some comfort guaranteed to the mother and the baby would not be fussy during the day, in this instance marketing the product in relation to crying.

*Being a young working mom who is still breastfeeding is quiet challenging... Well, not just for you but also for your little one as well as the person taking care of your little you while you are at work. @philipsaventsa Natural bottle makes it all easy, for these bottles mimic the breast for easier combination feeding. That way you know your little heart is not fussy all day while you are at work. Oh and the very same containers you use to store your expressed milk can easily be turned into a Natural bottle. Easy feeding without pouring and re-pouring, ensuring hygiene for your little heart #breastfeeding #naturalbottles #youngmom #breastmilk #expressmilk #babyboy #babysedi #lesedi #avent #philipsavent #babies #bottles #naturallatch #anticolic #ultrasoft* (Azwi Rambuda)

This post promoted combination feeding as a solution for the “challenges” that come with breastfeeding. The fact that the influencer mentioned expressing breastmilk and used the hashtag #expressmilk as opposed to promoting infant formula explicitly does not detract from the frame claiming bottle equivalency to the breast.

The parents and caregivers of children who cry a lot were influenced to use pacifiers and infant formula was subtly advertised through the marketing of bottles. While there was no direct marketing of infant formula, the implication that bottles and pacifiers were as good as breasts since they are “shaped like the breast” undermined the unique benefits of breastfeeding.

### ‘Do it your way’

In a different post, Mpoomy Ledwaba shared an image of herself breastfeeding, and highlighting the benefits of breastfeeding to her followers. However, this post was sponsored by NUK, a company which produces BMS. In her caption, she directed her followers to the NUK Instagram page and encouraged them to follow the page in order to stand a chance of winning a hamper by NUK.

*Antibodies, vitamins, trace elements – and a lot of love: breast milk has everything your child needs for healthy development in the first months” The best breastfeeding advice I can give you is: do it your way and with love. I’m giving away a @nuk_southafricahamper to one amazing mama, all you have to do is follow @nuk_southafricaand share your unique breastfeeding journey and if you are pregnant, what you look forward to. (*Mpoomy Ledwaba)

This is an example of marketing of BMS that is not very apparent but may be considered a contravention, as NUK also makes bottle and teats and directing followers to their page may be regarded as promotional practise. In addition, the ‘do it your way’ mention is one often embraced by BMS companies as a way to underplay the hygiene and health risks of using their products.

### Exciting (early) milestones

A more apparent advert of BMS was made by Nkateko Dinwiddy, who advertised baby food produced by Ella’s kitchen. The image shared was of a baby being spoon-fed solids and the packaging on the food packet showed that the food was suitable for infants aged four months and beyond. The caption on the image read:

*We’ve reached another exciting milestone for Suri as she’s now started her weaning journey. I remember having a lot of fun and making a lot of mess with Sana when she was weaning, but I remember finding it a little daunting too. Knowing when to start, what to start and how to keep it fun! That’s why I’m really happy to announce that I’ve partnered with @ella’skitchenuk to tell you about WEANSURY- a brilliant new online hub filled with lots of helpful information, top tips form the experts, recipes, and so much more to support you on your weaning journey – Nkateko Dinwiddy*

In this post, Nkateko Dinwiddy shared that she was weaning her child and had partnered with a BMS company called Ella’s Kitchen. In the post, she directed her followers to the company’s website to get more infant feeding advice. In this image, BMS is marketed targeting infants less than six months, which violates both the Code and R991. The food packets advertised in the image are marked “from 4 months”, implying that the influencer is marketing to mothers or caregivers of infants under six months old. At the time the image was posted, her infant was less than six months old, therefore the image idealised feeding solids to infants under six months of age. Suggesting that introducing semi-solids before six months is an ‘exciting milestone’ directly contradicts WHO EBF guidelines.

### Influencer framing of breastfeeding in relation to crying and sleeping

Another aim of this study was to analyse the posts on breastfeeding and how this is framed by the influencers on Instagram. From the posts analysed, 57% were images of influencers breastfeeding their children and the subsequent captions accompanying the posts encouraged their followers to practice the same. Influencers who advocated for breastfeeding highlighted how breastfeeding was ideal to calm a crying baby and to aid with sleep patterns. There was no evidence of sponsorship by companies or breastfeeding promotion organisations, e.g. GrowGreat or La Leche League, in these posts or associated hashtags.

During the annual breastfeeding week, the frequency of posts related to breastfeeding significantly increased by 43% among the influencers. In this period, most posts were about influencers promoting breastfeeding and highlighting its benefits. In the example below, the influencer shared her experience with tandem breastfeeding (nursing two babies at the same time) and the benefits of EBF. The caption on the image read;

*One of my favourite things about motherhood is breastfeeding, so I decided I would breastfeed Nuri till she’s 2 and even when I found out that I’m pregnant I was happy when my midwife told me its safe to breastfeed. However, our breastfeeding had reduced to just mornings and evenings but the closer we are to the arrival of our new baby the clingier Nuri has gotten yes I plan on breastfeeding both but it seems missy is super attached now. Moms who’ve breastfed more than one baby did you experience this? Or just clinginess towards the end of your pregnancy? #momtalk #breastfeedingmama #breastfeeding #breastfeedingmom #breastfeedingweek #postpartum #youngmum #breastfeedingbenefits #babies #mom #preggo #newmom #exclusivebreastmilk -Mpoomy Ledwaba*

Another example of a post from an influencer encouraging her followers to breastfeed is showcased in an image where the influencer was smiling and publicly breastfeeding and had the following caption:

*I breastfeed openly whenever and wherever Suri needs. I’ve learnt to block out the stares or the whispered comments form the non-approvers as I give my baby what she needs. My influence comes from my African upbringing where this approach to feeding babies was normalised by everyone around me. Don’t let anyone make you feel bad for what’s natural and normal. Enjoy your breastfeeding journey #normalisebreastfeeding #breastfeeding #breastfeedingmom – Nkateko Dinwiddy*

There were other posts promoting breastfeeding by the influencers and they shared their own personal journeys with breastfeeding. Despite none of the breastfeeding posts being sponsored, we found that posts encouraging breastfeeding accounted for the majority of infant feeding posts that were shared by the selected influencers.

There was little reference to breastfeeding in relation to crying or sleeping. Rather, some benefits to breastfeeding shared by the influencers were being nutritional, bonding between mother and child, and weight loss for the mother. The influencers who shared breastfeeding posts also highlighted some challenges that they encountered with breastfeeding. Some other influencers shared how their support systems did not agree with the concept of EBF and since they relied on them for taking care of their infants while they worked, their children were introduced to solids before turning six months old. Their followers responded sharing their own personal experiences with infant feeding, which is highlighted in the following section.

### Follower responses to influencer frames

It is worth noting that from this study, more infant feeding posts promoted breastfeeding (57%) as compared to those that promoted the use of BMS products (43%). However, as reported earlier in Table 1, the posts encouraging the use of BMS got as many likes and comments as the posts that encouraged breastfeeding. The framing of the followers’ responses to the BMS and breastfeeding posts were analysed to explore the degree to which audiences’ frames may align (or not) with Influencers. This is illustrated in Table 6.

**Table 6:** Follower’s framing of comments relating to posts initiated by the influencers

Influencer	Follower Comment Framing						Total	
	**Pro breastfeeding**		**Pro-BMS**		**Neutral/Other**			
	**N**	**%**	**N**	**%**	**N**	**%**	**N**	**%**
**Mpoomy Ledwaba**^**a**^	534	52.2	229	22.4	260	25.4	**1,023**	**5.6**
**Azwi Rambuda**^**a**^	100	29.4	150	44.1	190	55.9	**340**	**1.9**
**Jessica Nkosi**^**a**^	4	2.9	100	73.0	33	24.1	**137**	**0.8**
**Nkateko Dinwiddy**^**a**^	10,980	70.0	151	1.0	4 554	29.0	**15,685**	**85.6**
**Nandi Madida**	500	68.5	20	2.7	210	28.8	**730**	**4.0**
**Keke Mputhi**	235	59.6	5	1.3	154	39.1	**394**	**2.2**
**Ntando Kunene**	4	16.7	15	62.5	5	20.8	**24**	**0.1**
**Total**	**12,357**		**670**		**5,406**		**18,333**	**100**

^a^Influencers who were sponsored by BMS companies

### Influencer vs alternative frames in follower responses

There were examples of influencer posts and subsequent conversations outweighing a follower’s immediate social support system around the use of BMS, pacifiers in particular. As noted below, a follower mentioned that her grandmother was against the use of pacifiers and had advised she throw hers away. However, she was hardly sleeping at night and was reconsidering her decision following the conversations around the issue from the influencer’s post. Responding to Jessica Nkosi’s pacifier post, a follower had the following sentiments to share.

*Wow I had to throw the pacifiers in the bin because my son’s paternal granny is so against them and I’m also a neat freak so wouldn’t give my son something dirty. Now at night I hardly sleep cos he eats every 2 hours.*

In this case the mother’s sleep took precedence over the infant feeding method that was ideal for the baby. Responses of the followers from the described case and other similar posts indicated that the consensus among the influencers and their followers was that giving babies pacifiers was an effective way to calm a crying baby (and catch up on one’s own sleep).

In a video post, Ntando Kunene probed the opinions of her followers after her mother had suggested giving her four-month-old son solids. She was sceptical of her mother’s advice since she knew guidelines advised initiation of solids after six months of age. Responding to this, a follower mentioned how she had started her child on solids when she was hardly a month old. She gave the logic that the child cried a lot and after initiating solids, the crying decreased. From this comment, other people giving their opinions on the conversation highlighted how they found it helpful to introduce solids to children that had not even reached a month to deal with the excessive crying.

*… mine seems a bit crazy but my daughter started solids when she was hardly a month old , because she used to cry a lot even after the bottle so my mom suggested I start the solids. I felt it was waaaaayyy too soon but hey it worked*

Another response to the video was:

You waited this long? My grandmother waits for a month and then she starts feeding solids. These kids cry so much when they are hungry and sometimes milk alone doesn't do justice.

Some “experienced” mothers within the comments sections also opined that the recommended six months to introduce solids was unachievable, and that the mother would have lost their mind because of an excessively crying baby.

*I’m a mother of three...Start as soon as they start crying from hunger, you would know, 2 months and above but the books are shy to tell the truth ... they’ll be like 6 months ... at which point you would be at a mental institution*

A notable finding from this and other influencer-initiated posts was that influencers seldom responded to the questions posed by the followers. In the case of the video, Ntando Kunene did not comment or give feedback to her followers pertaining to the conversation that she had started. Due to this, it is unclear whether her inclination from the responses of her followers was more towards breastfeeding or not. This was not a sponsored post and no BMS product was advertised.

### Health frames by professionals on influencers’ posts

Despite regulations from the Code against it, some health professionals used the influencers’ platforms to give infant feeding advice. One such example is from a nurse who recommended the need for a baby to have a pacifier. In the comment, the nurse pointed out that some babies need pacifiers to soothe them even when not hungry, and that the pacifier helps with a good sleep at night. The comment read,

*I am a nurse, trust me I thought the same way before the baby arrived but reading more my anxieties were alleviated, some babies NEEEDDD a pacifier as they always want something to suck on even when they are not hungry, and it helps them sleep well at night not to mention prevention of sudden infant death.*

In this instance, a pacifier was portrayed as a preventative method against infant death by a health professional.

Despite the popularity of pacifiers among the influencers who posted them and their followers, some other responses to the pacifier posts were against their use, citing them as unsanitary. A follower’s response to a pacifier advert by an influencer highlighted how she was not using one because of the risk they pose on exposing children to diarrheal infections. Another follower responded that on doctor’s advice, she was not going to use a soother because she believed they are unhealthy.

Despite comments like these, the general slant in the responses were more positive towards what the influencers suggested, with some followers thanking the influencers for the suggestions on which pacifier to buy and how the pacifier helped soothe their own babies as seen in this quote: *“Just gave birth two weeks back and I'm planning to get one. Thank you @mrslitelu”.*

### Frames related to sleep and hunger

Belief that a child who is not well fed tends to sleep less and therefore the child’s diet needs to be complemented with BMS was popular among the followers. A follower mentioned that she started her child on solids at three months old, against the advice of health professionals; however it had turned out “great” for her;* “Started at 3 months cos she was just not getting full with the milk only… It turned out great though nurses discourage it.”*

## Discussion

### Extent and prominence of influencer posts’ coverage

Studies have shown that young mothers rely on social media, Instagram in particular, for advice, support and general information about breastfeeding and infant feeding methods [36]. Through Instagram influencers, content that creates discursive communities on various topics and themes related to breastfeeding is shared, and there is a high degree of interaction between the followers and the influencers [37]. The number of followers that an influencer has translates to the potential audience for these discussions and reach is established by the number of likes and comments a post receives [38].

From this study, the total number of followers of the 7 influencers that were analysed was 6,790,000. This reflects reach, the number of people who may have been directly exposed to the influencer infant feeding posts. As a ‘discursive community’ almost one million people directly engaged with the selected influencers through likes and 18,333 comments were made on the 62 infant feeding posts. As noted in the findings though, few influencers responded to follower comments, which suggests that the depth of discussion or discourse was relatively superficial. Unlike a study that suggests that micro and nano-influencers may engage more with their followers than macro influencers, [24] this type of pattern was not reflected in this study.

In this study, three companies were identified as sponsoring influencers to market BMS. These were Ella’s Kitchen, Phillips Avent and Nuk. Ella’s Kitchen sponsored Nkateko Dinwiddy to post BMS in the form of complementary foods for infants aged four months old. It is worth noting that the influencer involved is based in the UK, however, her followers are mainly South African, and through her post, they could access the website for Ella’s Kitchen and buy the products online. In the UK, regulations relating to the marketing of BMS may vary from those of South Africa; therefore, this could indicate the tactics that manufacturers use to market their products, in the process violating regulations. The other companies—NUK and Phillips Avent—did not explicitly market baby formula or solids, but rather marketed bottles and pacifiers; while pacifiers are not a contravention to the Code, the marketing of bottles and teats is regulated by the Code. This indicates awareness of regulations; however, companies still find ways to manoeuvre and market their products. Here we also note Giufferdi-Kahr and colleagues observation that influencers do not always disclose sponsorship and they may not even be aware of regulations [31].

BMS manufacturers take advantage of the wide audience reach the influencers have to market their products [10]. The South African influencers sponsored by BMS companies had a reach of 3,518,000 followers in this study. Other studies have found that BMS companies normally spend 10–15% of their gross profits to market their products in low and middle income countries [10]. However, with the advent of social media influencers, the reasonable assumption is that they spend less per post for the large audience that is commanded by the influencers when compared to traditional marketing methods, e.g. print advertisements [39]. This could follow that fewer resources are used in the marketing of BMS to target a significantly wider audience, making it cost effective [24].

Previous research has shown that Instagram has both negative and positive influences on its users in general [40]. Due to the large potential of social media influencers to influence behaviour, government bodies should increase monitoring of social media marketing to regulate the activities of the BMS manufacturers. In addition to unethical marketing, research has also shown that social media that focuses on breastfeeding can improve breastfeeding intentions, enhance knowledge pertaining to breastfeeding and can provide supportive communities among breastfeeding mothers [41]. These results are congruent with results from this study, in followers appreciated influencer breastfeeding posts.

### Violations of the code and R991

The Code, together with the local R991 prohibit the marketing of BMS for use as a total or partial replacement of breast milk [14]. A provision in the SA legislation is that employees of BMS manufacturers cannot contact members of the public to market their products, including via “internet sites”, which include social media platforms [26]. This study revealed that several provisions of the Code were violated, directly or indirectly, through manufacturer-sponsored posts that influencers shared. Four influencers directly advertised BMS products to their followers, portraying them to be best decisions that parents can make for their children in terms of assisting with problems of crying and sleeping. Even if some of the products were not defined as BMS in R991 or the Code, e.g. pacifiers, the same companies also produce BMS products that are covered in the regulations. Their sponsorship of influencers to draw followers to their websites, is an indirect form of BMS marketing.

In reference to a child who cries a lot, several influencers promoted the use of pacifiers. Within these posts, we found examples of followers reinforcing that this was effective in calming a crying baby, providing additional marketing support to the BMS companies. In the case of this research, some influencers posted adverts for pacifiers specifically designed for infants from birth to six months old. Despite this not violating R991 and the Code, this contravenes the UNICEF and WHO Guidelines for the Compliance for Advertising in Baby Friendly Healthcare facilities, which prohibits marketing pacifiers and nipple shields and regards the marketing of these as unacceptable [42]. This highlights ambiguity between what healthcare facilities accept and what happens in social media spaces.

While physiological factors influence a woman’s decision to breastfeed, societal factors have been observed to play a bigger role on a woman’s infant feeding decisions [40]. Recent studies have shown that breastfeeding mothers use social media for social support, seeking advice and as a source of information [21, 22]. Instagram influencers are known to create information sharing societies with their followers and BMS manufacturers use this to their advantage when marketing their products through paying influencers to endorse their brands [22]. The discursive communities created on social media through interactions of influencers and followers in the comments section have the potential of reinforcing social norms that undermine breastfeeding on posts that promote the use of BMS or related products. For example, seeing an influencer using a pacifier to help her baby sleep, and giving testimonials of how this has made her life easier, as in the post by Jessica Nkosi, might encourage her followers to do the same; they might also visit the company website, which also markets infant formula. This arguably contravenes provision 7 of the Regulation 991, stating that no person shall undertake or participate in any promotional practices in respect of BMS [13].

### The use of framing techniques to influence behaviour

Framing theory suggests that the way something is presented to an audience influences the choices that the audience makes pertaining to how they process the information, and can be viewed as a form of agenda setting [35]. From the posts and comments that were analysed for this study, it is clear that influencers marketing BMS products adopted similar framing techniques that have been used by industry in other contexts to increase social acceptance and desirability, including the use of metaphors, catch-phrases, and stories.

According to the framing theory, metaphors are used when an idea or concept is compared to something else in order to create desirability [43]. An example of the use of metaphors from this study was when pacifiers and teats were compared to the breast. Specifically, on several posts, two influencers mentioned how the NUK pacifier and the Phillips Avent teat mimic the breast and were suitable for infants from as young as 0–6 months in the case of the pacifier. A study on whether breastfeeding babies should be given pacifiers found that early introduction of pacifiers may lead to “nipple confusion” and may lead to incorrect latching, both which undermine breastfeeding [44].

Framing theory also suggests that the use of catch-phrases make a message more memorable and relatable [43]. In the language of social media, catch-phrases are sometimes presented in the form of hashtags, and they make content easier to find [45]. All of the posts that were analysed for this study contained at least one hashtag to improve the reach of the posts. Examples of when hashtags were used in the promotion of breastfeeding included #breastfeeding and #normalizebreastfeeding. However, in the adverts, BMS manufacturer companies were hash-tagged to increase visibility of the posts. Examples include #PhillipsAvent and #naturalbottles. The use of hashtags and the catch-phrases from within the hashtags increases the volumes on the posts. In addition, using company hashtags could bring Instagram followers to the main company websites, where other BMS products are marketed. Such catch-phrases, when used to market BMS, can have detrimental effects on the efforts to promote ideal infant feeding practises [45].

Through story-telling, a topic is framed in a vivid and memorable way to the effect that an audience can be drawn to it [43]. Influencer posts analysed in this study indicated that influencers use this technique through captioning their posts with a personal experience using BMS products on sponsored posts or breastfeeding on the non-sponsored posts. According to the theory, depending on how well the story is told through the captions or through images and videos, the targeted audience is likely to follow the recommendations from the posts [40]. The visuals of influencers breastfeeding and the support they receive while doing so captured follower attention, as seen through high levels of engagement.

In this study, there was a high level of engagement on the posts promoting breastfeeding and followers were encouraged to practise the same. Despite the common belief that breastfeeding is practised by people of low socio-economic status [46], when the followers saw people they look up to breastfeeding, many expressed motivation to copy the behaviour in their comments. The impact of marketing could therefore be beneficial to promote infant feeding according to regulations and guidelines by health authorities.

### Implications of violations

Even though the data analysed in this study did not show direct advertisement of infant formula or porridges, there was clear use of calculated marketing techniques by companies, which did not make the marketing apparent. This was done through marketing products used to facilitate administration of apparent BMS, like feeding bottles. While this may indicate the effectiveness of the Regulation 991 in reducing direct marketing of infant formula, conclusions can be made that companies producing BMS are aware of the regulations of the Code, but still find ways of advertising their products.

The use of BMS company hashtags was a subtle way of directing followers to products that are otherwise restricted from being advertised. This finding resonates with other literature from a study looking at marketing of BMS in South Africa, which made the same conclusions [26]. According to both the Code and R991, the penalties that can be imposed for violating regulations include fining or imprisonment or both to companies found in violation [13]. However, to date there has not been any published information to that effect despite the violations by the manufacturers.

### Study limitations

While the posts analysed received almost a million likes and about eighteen thousand comments, reflecting reach, the sample size for the influencers analysed was relatively small (a total of 7). We might have introduced bias through the influencer selection methods used, as there was no central repository of Instagram influencers available. This is a methodological area in social media research that needs further attention. Restricting the sample to South African influencers does not reflect non-South African influencers who South Africans may be following, meaning that this study likely underestimates how BMS companies may be using influencers in the South African context. With the use of Instagram, the influencer who makes a post is at liberty to delete a post after they have made it, such that some posts may have been deleted after engagement with the public and potential influence has already been made. This was the case with the one video that was analysed as a part of this study. We had however downloaded it and its subsequent comments before it was deleted. In addition to this feature on Instagram making monitoring of regulation violations more challenging, it also may influence the replicability of this type of study. The use of Instagram as compared to Facebook may also be another limitation of this study, as Facebook has more users than Instagram. We also acknowledge that this study did not directly engage with followers and relied on research from other contexts to infer the possible impact of marketing. As in other contexts, we need studies that can quantify the degree to which exposure to social media channels influences consumer behavior.

## Conclusions

The tendency for followers to agree with the recommendations of the influencers, whether breastfeeding of BMS, aligns with literature that suggests that direct marketing by social media influences followers, though our study did not confirm this independently. Observation of online interactions around the posts also reinforces the conceptual framework in Fig. 1, suggesting that when there is limited enforcement of regulations and guidelines, as is the case with South Africa’s R991, public marketing of BMS may result in creation of attitudes and social norms that favour BMS [47]. The absence of direct marketing of commercial infant formula by the influencers selected for this study suggests that there is knowledge of the regulation against marketing of BMS by manufacturers, but they still find a way of marketing other products that are linked to BMS and which undermine breastfeeding.

This study identified Instagram as a channel of influence for infant feeding in South Africa. BMS marketing on social media is cause for concern that requires closer monitoring and regulation, particularly as regards violations of the Code and R991.There should be a section in the national legislation that deals specifically with the marketing of BMS on social media. In addition, BMS manufacturers should take responsibility for their marketing practises on social media platforms, such as Instagram, as part of their responsibility to comply with national legislation. Influencers also must be educated on doing research before endorsing brands, especially when it comes to health and specifically infant health.

Despite evidence of the unethical marketing of BMS on social media, the channel of Instagram itself should not be disregarded as a mechanism to promote breastfeeding. As the world is moving towards a more digital era, public health communication strategies should shift focus from traditional print messaging to social media communication. Specifically, there is need to explore using influencers as messengers. Using platforms like Instagram, where the responses from the audience can be accessed, can also be an advantage because misconceptions can be addressed and corrected given the interactive nature of the platforms. In South Africa, cumulatively, eight million people have access to all social media platforms [48]. It is time for public health communication experts to design more strategic social media strategies to promote EBF and counteract the marketing of BMS companies.

## Data Availability

The datasets used and/or analysed during the current study are available from the corresponding author on reasonable request. URLs to the posts directly quoted in the manuscript have been included.

## Abbreviations

BMS: Breastmilk substitutes
EBF: Exclusive breastfeeding
ICMBMS: International Code for the marketing of breastmilk substitutes
IYCF: Infant and young child feeding
R991: Regulation 991
WHA: World Health Assembly
WHO: World Health Organization
